# DNA Aptamers: Reloaded Tools for Breast Cancer Therapeutics

**DOI:** 10.3390/cancers18050766

**Published:** 2026-02-27

**Authors:** Karen Carrasco-Maure, Mauricio González-Olivares, Lorena Lobos-González

**Affiliations:** 1 Centro de Estudios en Ejercicio, Metabolismo y Cáncer (CEMC), Interdisciplinary Nucleus of Biology and Genetics (NiBG), Instituto de Ciencias Biomédicas (ICBM), University of Chile, Santiago 8370854, Chile; 2 Advanced Center for Chronic Diseases (ACCDIS), Santiago 8330024, Chile; 3 Faculty of Chemical and Pharmaceutical Sciences, Universidad de Chile, Santiago 8380494, Chile

**Keywords:** aptamers, breast cancer, diagnostic platforms, aptasensors, liquid biopsy, targeted therapy, theranostics, SELEX technology, nanomedicine, cancer biomarker

## Abstract

Breast cancer continues to be a major challenge in global health, in part due to significant inequalities in access to costly diagnostic and therapeutic technologies based on antibodies. Their manufacturing requires complex and expensive bioproduction systems, resulting in limited availability of these tools—essential for early detection and targeted treatment—in many regions, particularly in Latin America. This gap has highlighted the need for cost-effective and scalable theranostic alternatives, increasing interest in aptamers. Obtained through SELEX technology, aptamers are synthetic DNA or RNA oligomers that fold into functional structures. Among their advantages are high affinity for their target, low immunogenicity, and chemical synthesis, which assures reproducible production. Aptamers have expanded the landscape of diagnostic platforms through the development of sensitive aptasensors, liquid biopsy strategies, and imaging systems based on nanomedicines. They also contribute to targeted therapy by recognizing cancer biomarkers selectively and enabling controlled drug delivery. This review presents a critical summary of advances in aptamer-based theranostics for breast cancer, addressing molecular mechanisms, structural folding, selective ligand binding, and nanomaterial interfacing. We also discuss applications in extracellular vesicle capture, cancer stem cell detection, and therapeutic conjugates, emphasizing their advantages and limitations relative to approaches based on antibodies. Overall, current advances show aptamers as emerging tools capable of democratizing precision oncology, particularly in regions where access to advanced technologies remains limited.

## Simple Summary

Breast cancer is still a leading cause of mortality among women, mainly due to late detection, tumor heterogeneity, and limited access to advanced therapies. Therefore, there is a need for precise, reproducible, and affordable tools for diagnostics and therapeutics. Aptamers—synthetic single-stranded oligonucleotides that fold into specific three-dimensional structures—offer high affinity and selectivity for cancer targets, with advantages such as low immunogenicity, chemical stability, and scalable synthesis. This review summarizes recent progress in the use of aptamers for breast cancer, including biosensors and liquid biopsy applications, prognostic profiling of aggressive subtypes, and therapeutic conjugates such as aptamer–drug and aptamer–siRNA combinations. Upon overcoming current challenges in pharmacokinetics and clinical validation, aptamers should become transformative tools for breast cancer by enabling precision oncology and broad accessibility.

## 1. Introduction

Breast cancer is the most frequently diagnosed neoplasm in women and one of the leading causes of mortality related to cancer worldwide. In 2020, more than 2.3 million new cases and approximately 685,000 deaths were reported, figures that continue to rise and reflect both population growth and persistent disparities in access to diagnosis and treatment [1,2]. The disease encompasses a heterogeneous spectrum of molecular subtypes, such as luminal A, luminal B, HER2-positive, and triple-negative, whose biological differences determine their aggressiveness, therapeutic response, and prognosis [3]. Despite advances in targeted therapies and immunotherapies, access to high-cost drugs is still limited, especially in contexts where economic inequalities restrict the implementation of personalized medicine strategies. This gap between scientific development and clinical availability has created an urgent need for tools that are more accessible, reproducible, and versatile and that can be integrated into early detection and selective treatment programs.

In this context, aptamers have emerged as a promising alternative to conventional antibodies. These are DNA or RNA oligonucleotides selected through the SELEX process (Systematic Evolution of Ligands by EXponential enrichment), capable of folding into specific three-dimensional structures that recognize molecular targets with high affinity and specificity [4,5]. Unlike proteins, aptamers can be produced by chemical synthesis, allowing for precise control over their composition, high batch-to-batch reproducibility, and substantially lower costs. Additionally, their chemical modifications—such as 2′-O-methyl substitutions, phosphorothioate linkages, or polymer conjugation—enhance their stability against nucleases and prolong their circulating half-life [6,7]. These characteristics led them to be considered akin to “synthetic antibodies” and to explore their diagnostic and therapeutic potential, especially in oncology, where molecular precision and cost-effectiveness are convergent priorities.

The growing body of evidence demonstrates that aptamers can transform clinical practice in breast cancer by combining analytical sensitivity, molecular specificity, and technological scalability. In the diagnostic field, electrochemical, optical, and magneto-spectroscopic biosensors have been developed that can detect tumor biomarkers such as HER2, MUC1, EpCAM, and PD-L1 with high sensitivity and minimal invasiveness [8,9]. For prognosis, they enable the identification of aggressive subpopulations and extracellular vesicles associated with recurrence or therapeutic resistance [10], while in therapy, aptamer–drug conjugates, aptamer–siRNA conjugates, and theragnostic platforms have demonstrated antitumor efficacy with reduced systemic toxicity [11,12]. Nevertheless, challenges remain: their in vivo half-life is still limited, clinical validation is still incipient, and the transition to controlled studies faces regulatory and economic obstacles. Understanding and overcoming these limitations is essential to establish aptamers as next-generation clinical tools, capable of reducing disparities in breast cancer diagnosis and treatment on a global scale. Within this framework, the present review seeks to integrate and critically analyze the available evidence on the use of aptamers in breast oncology, highlighting both their experimental robustness and the opportunities for translational development that could make them a feasible, economical, and precise strategy for early detection, prognostic monitoring, and targeted therapy of breast cancer.

### 1.1. Aptamers: Development and Advantages

**Figure 1:**
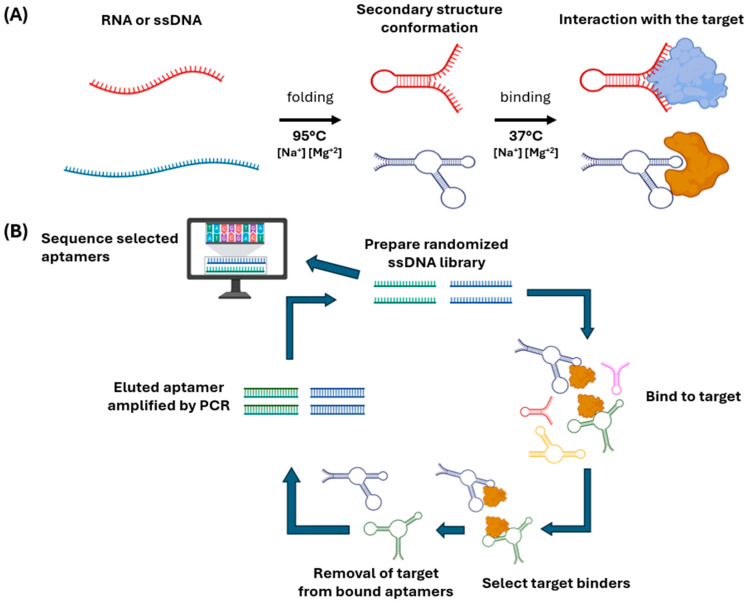
Basis of aptamer function and iterative selection process to obtain aptamers. (**A**) The general mechanism of action of aptamers, which can be single-stranded RNA or DNA (ssDNA) molecules, is shown. When subjected to appropriate conditions, these sequences adopt secondary and tertiary structures defined by intramolecular folding processes, forming loops, hairpins, and double-stranded regions. These three-dimensional conformations allow them to recognize and bind specifically to a target molecule—a protein, peptide, or small metabolite—or even a whole cell. (**B**) The iterative selection process called SELEX (systematic evolution of ligands by exponential enrichment), used to obtain aptamers with high affinity for a target molecule, is outlined. A library of random DNA or RNA sequences is folded and incubated with the target molecule, the library being in excess of the target to select the sequences with the highest affinities. Aptamers that do not bind are eliminated in the separation stage, and aptamers bound to the target are then eluted and subsequently amplified by PCR to generate a new population enriched in the higher-affinity aptamers. This cycle of selection (binding, separation, elution) and amplification is repeated multiple times until a pool of aptamers with high specificity and affinity is obtained. These aptamers are sequenced, analyzed with bioinformatics tools, synthesized, and characterized individually [5,13,14].

Aptamers are single-stranded DNA or RNA oligonucleotides capable of recognizing a wide range of targets with high affinity and specificity, from small molecules to proteins, cells, and even entire tissues, as illustrated in Figure 1A. Their discovery in the early 1990s by Tuerk and Gold, and independently by Ellington and Szostak, marked a milestone in molecular biology by demonstrating that random RNA sequences could acquire molecular recognition functions through an in vitro selection process known as SELEX (Systematic Evolution of Ligands by EXponential enrichment) [4,5]. This method, based on iterative cycles of binding, separation, and amplification, allows for the isolation of sequences able to fold into specific three-dimensional structures to interact with their target, as outlined in Figure 1B. Each selection cycle increases the enrichment of higher-affinity sequences, generating an optimized population of nucleotide ligands. Over time, SELEX has diversified into variants that incorporate physiological conditions (cell-SELEX, tissue-SELEX, in vivo-SELEX) or chemically modified libraries that expand the stability and structural diversity of aptamers [6]. Nevertheless, the overall efficiency of the process remains a technical challenge: comparative studies estimate success rates below 1% from the initial libraries, and effective enrichment critically depends on target purity, selection conditions, and amplification parameters [10].

### 1.2. Advantages of Using Aptamers as Clinical Tools

The molecular recognition of an aptamer is based on its three-dimensional architecture. These molecules can adopt stable secondary and tertiary structures—such as hairpins, pseudoknots, i-motifs, or G-quadruplexes—that generate specific cavities and contact surfaces. The interactions with the target include hydrogen bonds, base stacking, electrostatic and hydrophobic forces, allowing discrimination between isoforms or even variants with a single amino acid change [15,16]. Critically, this structural specificity does not depend on an immune response, which distinguishes aptamers from conventional antibodies and grants them low immunogenicity; a broader comparison of aptamers versus alternative targeting modalities is provided in Table 1. Added to this are chemical modifications at the nucleotides or at the 3′ and 5′ ends—such as 2′-O-methyl substitutions, phosphorothioate linkages, PEG conjugation, or metal anchoring—that increase nuclease resistance and prolong plasma half-life up to eight hours in animal models, enhancing structural stability without compromising affinity or selectivity [7,17]. These adaptations confer aptamers with functional versatility that goes beyond their role as experimental probes, allowing their integration into advanced diagnostic and therapeutic platforms.

Compared to monoclonal antibodies or other protein-based therapeutic platforms, aptamers offer substantial advantages. Their smaller molecular size (10–30 kDa versus ~150 kDa for an IgG) facilitates tissue penetration and access to sterically restricted epitopes [26]. Chemical synthesis eliminates the need for cell cultures, reduces batch-to-batch variability, and simplifies large-scale production, lowering costs and development times [27]. Additionally, their thermal stability and ability to regenerate after denaturation allow for their reuse in detection platforms and repeated assays without loss of activity. Typical binding affinities range from nanomolar to even picomolar, comparable to or greater than those of monoclonal antibodies, which strengthens their potential for high-precision clinical applications [15]. However, their limitations should be recognized: rapid renal clearance of small molecules (<30 kDa), susceptibility to degradation in complex biological matrices, and limited comparative validation in clinical trials still restrict their routine implementation. In summary, advances in SELEX selection and chemical engineering have transformed aptamers into a robust molecular platform capable of combining the specificity of antibodies with the synthetic flexibility of oligonucleotides. Their development marks a convergence between structural biology and applied nanotechnology, offering a solid foundation for strategies of early diagnosis, prognosis, and targeted therapy in breast cancer. Nonetheless, full clinical translation of this technology will rely on optimizing their stability in vivo, standardizing selection protocols, and validating their performance in comparative clinical models that demonstrate tangible advantages over protein-based therapies.

## 2. The Use of Aptamers in Breast Cancer Diagnostics

**Figure 2:**
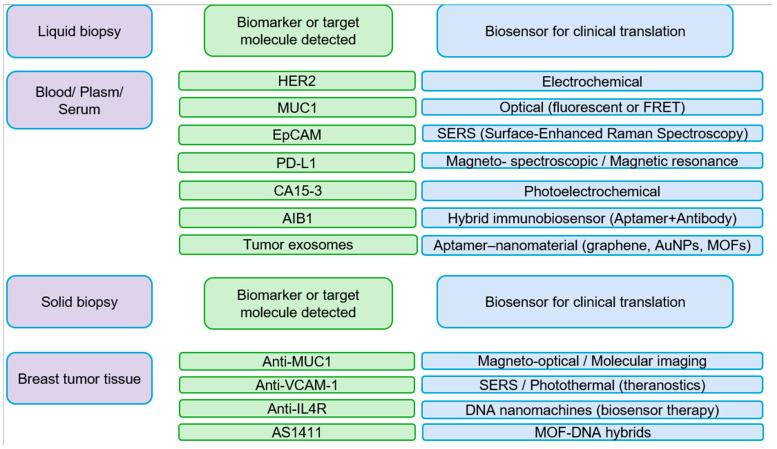
Aptamer-based biosensing platforms for biomarker detection in liquid and solid biopsies of breast cancer. Overview of aptamer-guided biosensors classified according to the biological source and the detection principle. In liquid biopsies (blood, plasma, or serum), aptamers are used to identify circulating biomarkers such as HER2, MUC1, EpCAM, PD-L1, CA15-3, AIB1, and tumor-derived exosomes through electrochemical, optical, SERS, magneto-spectroscopic, or hybrid aptamer–nanomaterial systems. In solid biopsies (tumor tissue), aptamer-conjugated platforms enable highly specific imaging and theranostic approaches targeting molecules such as MUC1, VCAM-1, IL4R, and nucleolin (AS1411), employing magneto-optical, photothermal, DNA nanomachine, or MOF–DNA hybrid technologies. Collectively, these strategies exemplify the versatility of aptamers as molecular recognition elements in diagnostic and therapeutic biosensing for breast cancer [29,30,31,32,33,34].

Early detection of tumor biomarkers is one of the most effective strategies to reduce mortality associated with breast cancer. Aptamers, thanks to their high affinity, molecular specificity, and ease of chemical modification, have established themselves as versatile tools in the development of diagnostic platforms capable of identifying proteins, cells, and tumor vesicles with sensitivity comparable to that of monoclonal antibodies. Their thermal stability, low cost, and ability to be regenerated after multiple cycles of use make them ideal candidates for reusable and low-cost clinical devices. In the oncology field, aptamers have been designed to recognize clinically relevant biomarkers such as HER2, MUC1, EpCAM, PD-L1, and nucleolin and have been integrated into biosensors, liquid biopsy techniques, and even high-resolution molecular imaging systems [28,29]. These applications and representative detection formats are schematically depicted in Figure 2.

### 2.1. Aptamer Sensors in Solid Matrices

The ability of aptamers to recognize biologically relevant molecules with high affinity and specificity makes them central elements in a new generation of biosensors applied to breast cancer diagnostics. These DNA or RNA molecules are designed to bind to key targets such as the HER2 receptor [30], the AIB1 protein amplified in breast cancer [31], and the adhesion molecule EpCAM, used to capture and visualize circulating tumor cells [32]. In recent years, selection strategies based on genomic analysis and artificial intelligence have made it possible to identify aptamers against complex targets, optimizing their secondary structure and affinity using predictive models [35]. These advances have strengthened the sensitivity and specificity of aptameric biosensors, which can detect biomarker concentrations in the pico- or femtomolar range, surpassing the detection limits of conventional techniques like ELISA or immunohistochemistry.

The incorporation of nanomaterials and noble metals has significantly enhanced the analytical performance of biosensors. The conjugation of aptamers with superparamagnetic nanoparticles, silver–gold hybrids, or graphene-based systems has made it possible to amplify signals and obtain high-resolution images through magnetic resonance imaging or surface-enhanced Raman spectroscopy (SERS) [28,29]. These devices combine precise molecular recognition with advanced optical and magnetic properties, enabling the simultaneous detection and quantification of multiple biomarkers in a single test. Hybrid biosensors with structures such as Fe_3_O_4_@Au–graphene demonstrate high conductivity, improved stability, and reduced interference from serum components, which increases reliability in complex biological samples [13]. This technological integration has given rise to theranostic platforms, capable not only of diagnosing but also of guiding or activating localized treatments under optical or magnetic stimuli.

Beyond passive detection, some advanced design systems have demonstrated the possibility of coupling molecular recognition with active immunological modulation. For example, the Aptamer–Biotin–Streptavidin–C1q complex can induce complement activation, paving the way for immunodiagnostic strategies with simultaneous therapeutic effects [36]. However, progress toward clinical application requires addressing limitations related to in vivo stability, variability in biological matrices, and inter-laboratory reproducibility, all of which are essential factors for standardizing sensitivity, specificity, and accuracy compared to conventional immunological methods.

Taken together, aptamer-based biosensors represent a significant evolution in the molecular detection of breast cancer by integrating highly specific recognition, multifunctional response, and compatibility with emerging technologies. Their development marks a transition from analytical diagnostics toward intelligent systems capable of combining detection, monitoring, and therapeutic response in a single platform.

### 2.2. Approaches Based on Aptasensors for Liquid Biopsies and Early Detection of Breast Cancer

Liquid biopsies, which analyze tumor components in blood and other bodily fluids, have advanced significantly thanks to the use of aptamers, establishing themselves as a minimally invasive method for the diagnosis and monitoring of breast cancer. Due to their high affinity and specificity, aptamers are ideal tools for capturing circulating tumor cells (CTCs), exosomes, and serum biomarkers associated with metastatic progression. Strategies combining aptamers with magnetic beads or quantum dots enable the detection and quantification of CTCs in MCF-7 lines with high sensitivity and a reduction in false positives [37], while techniques like dual rolling circle amplification and multiplexed electrochemical biosensors further improve the detection limit [19,38]. Although the detection levels achieved by these platforms are comparable to those of conventional immunoassays, clinical validation remains limited, and outcomes depend on the aptamer’s stability and serum composition.

Optical and electrochemical biosensors that integrate aptamers and silver nanorods, graphene, or Fe_3_O_4_, have been shown to detect tumor cells in blood rapidly and reproducibly, offering high conductivity and low interference [39,40]. Similarly, electrochemiluminescent and photoelectrochemical technologies identify SK-BR-3 and MCF-7 cells with great specificity, expanding the potential of molecular diagnostics in portable and microfluidic systems [41].

As for the analysis of extracellular vesicles, magnetic nanocomposites functionalized with aptamers have been developed to isolate PD-L1^+^ exosomes, analyzed via surface-enhanced Raman spectroscopy (SERS), enabling precise discrimination between tumor samples and controls [9]. Likewise, an RNA aptamer targeting breast cancer exosomes has demonstrated high-resolution optical and electrochemical applications [42]. In parallel, aptamer-based immunosensors combined with metallic or carbon nanoparticles simultaneously quantify CA15-3, HER2, and MUC1, showing significant correlation with clinical levels [9,30]. Specific sensors for MUC1 coupled to AuNPs or graphene–Fe_3_O_4_ hybrid materials improve stability and sensitivity [39,40].

Overall, these approaches establish aptamers as pillars of next-generation liquid biopsies, capable of integrating capture, detection, and molecular analysis into a single system. Nonetheless, their clinical translation requires standardizing protocols, evaluating biological interferences and confirming sensitivity and specificity in longitudinal studies—necessary conditions for establishing their routine use in early breast cancer diagnosis.

### 2.3. Aptamers as Targeting Agents for Tumor Imaging

**Figure 3:**
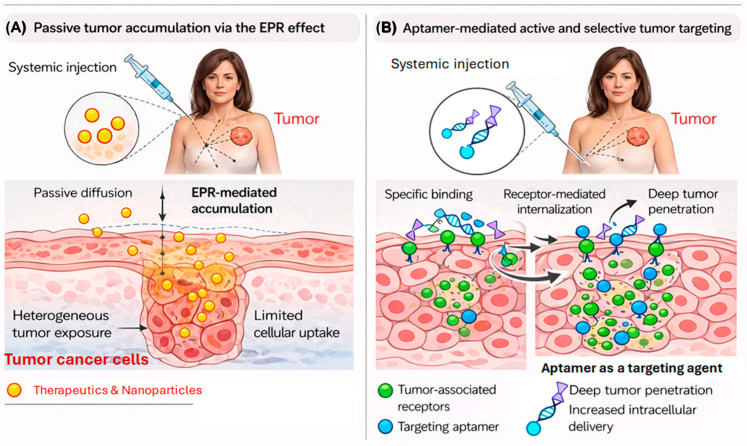
Comparative schematic of targeting strategies in molecular imaging and therapy. (**A**) Conventional delivery systems rely mainly on passive diffusion and the enhanced permeability and retention (EPR) effect, resulting in non-specific accumulation of imaging or therapeutic agents within tumor tissue. (**B**) Aptamer-functionalized systems provide active molecular recognition through high-affinity ligand binding, guiding nanoparticles or contrast agents directly to tumor-associated receptors. This “aptamer-as-a-target-enhancer” strategy improves localization, imaging contrast, and therapeutic precision, exemplifying the transition from passive to actively targeted nanomedicine in breast cancer [43,44,45,46]. (Part of the image was created by the author using BIORENDER).

In molecular imaging and targeted drug delivery, the addition of aptamers to conventional nanosystems transforms their performance from passive accumulation to active, ligand-guided targeting. While traditional delivery systems rely mainly on the enhanced permeability and retention (EPR) effect, aptamer-functionalized constructs actively recognize tumor-associated receptors, guiding imaging probes or therapeutic cargos directly to malignant cells. This molecular precision enhances contrast resolution, reduces off-target distribution, and enables the development of theranostic platforms capable of simultaneous diagnosis and therapy. As illustrated in Figure 3, aptamers act as targeting enhancers, improving the specificity and efficiency of nanoparticle-based imaging and treatment strategies in breast cancer compared to conventional non-targeted systems.

In the field of molecular imaging applied to breast cancer, aptamers are used as highly selective ligands capable of directing contrast agents toward tumor cells, increasing spatial resolution and diagnostic specificity. The conjugation of superparamagnetic iron oxide nanoparticles with anti-VCAM-1 and anti-IL4R aptamers has enabled high-definition imaging via magnetic resonance, in addition to enabling a theranostic approach that combines diagnostics and targeted treatment [29]. Complementarily, perfluoropolyether nanoparticles enriched with fluorine-19 have been designed to generate dual images through magnetic resonance and optical imaging, while anti-MUC1 aptamers have demonstrated efficacy in single-photon emission computed tomography (SPECT) in triple-negative breast cancer models, expanding the clinical applicability of these hybrid platforms [33,40]. These strategies highlight the ability of aptamers to selectively direct molecular contrast agents and improve tumor delineation without the need for conventional radioactive markers.

In the area of photoinduced imaging and photothermal therapy, aptamers conjugated with bimetallic silver and gold structures have enabled specific detection via surface-enhanced Raman spectroscopy (SERS) and at the same time facilitated localized destruction of tumor cells under infrared irradiation [28]. Similarly, nanocomplexes functionalized with the AS1411 aptamer have been evaluated as dual-imaging and photothermal treatment platforms, demonstrating significant reduction in tumor growth and high cellular specificity [34]. These systems represent an example of effective theranostics, in which the same molecule acts as sensor, guide, and therapeutic agent, minimizing off-target toxicity.

The most advanced approaches incorporate DNA nanomachines conjugated with gold, activated by endogenous mRNA, allowing in situ imaging along with a highly precise synergistic therapeutic response [47]. Likewise, aptamers are being explored for photoelectrochemical detection of cancer cells and for imaging via sequential emission computed tomography (SECT) in animal models of aggressive breast cancer [33,41]. Although these systems show significant progress towards integrating diagnosis and therapy in a single vector, their clinical translation still requires validation of parameters such as biodistribution, in vivo stability, and long-term biocompatibility.

Overall, the incorporation of aptamers into molecular imaging technologies represents a decisive advance toward precision medicine, allowing the specific localization of lesions, non-invasive monitoring of therapeutic response, and the possibility of combining diagnosis and treatment within a single construct. However, most developments remain in the preclinical phase, and clinical implementation will depend on optimizing the pharmacokinetics of the conjugates and establishing standardized protocols that ensure sensitivity and safety equal to or surpassing those of conventional imaging platforms.

## 3. Aptamers Used in the Prognosis of Breast Cancer

Accurate prediction of breast cancer progression is fundamental for guiding therapeutic decisions and optimizing clinical management. In this context, aptamers have emerged as powerful prognostic tools capable of detecting biomarkers associated with tumor aggressiveness, recurrence risk, and therapeutic resistance. By specifically recognizing molecular targets implicated in oncogenic signaling, epithelial–mesenchymal transition, and metastatic dissemination, aptamers provide both qualitative and quantitative information that supports patient stratification and personalized treatment planning. Their ability to discriminate subtle molecular variations in functional proteins, cell surface receptors, or extracellular vesicle cargos positions them as promising analytical and translational instruments for improving prognostic precision in breast cancer management.

### 3.1. Biomarkers for Quantifiable Prognosis

A prominent example is the CA15-3 antigen, a classic serum marker whose ultrasensitive quantification using an aptamer-based FRET immunosensor has enabled dynamic monitoring of tumor burden and therapeutic response [39]. Similarly, electrochemical biosensors targeting HER2 identify SK-BR-3 cells with great precision [30].

Beyond static detection, colocalization-activated DNA assemblies allow visualization of HER2 dimerization, a phenomenon that reflects receptor activation and correlates with an unfavorable prognosis [47]. Additionally, photo-crosslinkable aptamers against ERBB3 detect its association states, which are implicated in therapeutic resistance [48]. These approaches provide a superior level of molecular and temporal resolution compared to immunohistochemistry (IHC) or qPCR, which require fixed tissue and do not report on the functional activity of receptors, thus offering a more dynamic readout of tumor biology.

These advances show how aptamers make it possible not only to measure the presence of biomarkers but also to assess their functional activity, providing a comprehensive diagnostic tool that can guide risk stratification and inform treatment in breast cancer patients.

### 3.2. Aggressive Subpopulations and Cancer Stem Cells

A major breakthrough is the ability of aptamers to identify aggressive subpopulations and cancer stem cells (CSCs), which are responsible for recurrence and chemoresistance. The cell-SELEX technology has enabled the generation of specific aptamers against CD133 and CD49c, which are used to detect and isolate CSCs in breast cancer [49,50,51]. In triple-negative breast cancer models, nanoparticles guided by anti-CD133 aptamers have been used to deliver therapeutic anti-miRNAs, reducing proliferation and invasiveness, thereby validating CD133 as a prognostic marker and functional therapeutic target [49]. Similarly, aptamers against CD49c can identify highly invasive phenotypes associated with lower survival rates [51]. These discoveries integrate the detection and modulation of high-risk subpopulations, going beyond the descriptive approach of conventional proteomics, though their clinical validation still requires direct comparisons with cytometric and histological assays in large cohorts.

The molecular detection and characterization of these aggressive subpopulations with aptamers enable more precise and personalized prognostic stratification. This advanced approach contributes to improving clinical prognosis, as it identifies patients at greater risk of progression and paves the way for targeted therapies that effectively attack cancer stem cells—crucial for preventing treatment resistance and tumor recurrence.

### 3.3. Multiligand Signatures and Prediction of Clinical Outcomes

Beyond individual biomarkers, aptamers facilitate the creation of multiligand molecular signatures with high prognostic value. The poly-ligand profiling technique uses aptamer libraries to assess multiple interactions and distinguish patients treated with trastuzumab according to their clinical course [52]. In parallel, mechanistic studies have identified nucleolin (NCL) as a key modulator of tumor aggressiveness. Targeting NCL with aptamers regulates oncogenic microRNAs involved in cell proliferation and migration, showing that blocking NCL serves not only for diagnosis but also modulates functional pathways related to prognosis [53]. Similarly, aptamers against the kinases MNK1b and VRK1 suppress protein translation and tumor proliferation, proposing these enzymes as indicators of poor prognosis and potential therapeutic targets [34,48]. These findings show how aptamers can causally determine the function of their targets, unifying detection and functional validation within the same experiment.

### 3.4. Oncogenes and Kinases with Prognostic Relevance

On the other hand, the PDGFRβ receptor, which is highly elevated in triple-negative tumors, has been identified as a relevant target for aptamers, whose inhibition has been shown to reduce lung metastasis and alter tumor–stroma interaction—factors related to increased patient survival [12]. Furthermore, an aptamer with affinity for the β subunit of ATP synthase located on the plasma membrane has been reported, proposed as an early marker of aggressive tumor phenotype with potential for therapeutic applications [47].

These findings underscore the ability of aptamers not only to detect and characterize proteins involved in oncogenesis and tumor progression but also to modulate their activity, opening new avenues for more precise prognosis and the development of personalized therapies in breast cancer.

### 3.5. Tumor Microenvironment and Immunosuppression

Finally, the role of the tumor microenvironment (TME) as a prognostic determinant has also been explored using aptamers. The quantification of PD-L1^+^ exosomes through SERS platforms functionalized with aptamers has enabled the correlation of immunosuppressive load with clinical progression [9]. Similarly, aptamers against PDGFRβ, which is overexpressed in triple-negative tumors, reduce the migration of mesenchymal cells and the formation of the metastatic niche, thereby modulating the tumor environment and improving survival [12]. Complementarily, aptamers that block the recruitment of CD4^+^ T lymphocytes reverse immunosuppression and enhance the antitumor response, correlating with a better prognosis [54]. In this regard, the connection between diagnosis and prognosis is evident: biomarkers such as HER2 or PDGFRβ, initially detected for diagnostic purposes, acquire prognostic significance when their activation or modulation with aptamers directly alters metastatic or immunological pathways.

Taken together, current evidence shows that aptamers provide a mechanistic perspective on breast cancer prognosis by integrating molecular detection, biological function, and therapeutic response. While many results remain at the preclinical stage, their ability to combine specificity, sensitivity, and functional analysis positions them as promising tools for prognostic stratification and precision medicine, complementing and even surpassing the potential of conventional tumor assessment methods. A comparative overview of these modalities (antagonism versus targeted delivery), including key targets and therapeutic cargos, is shown in Table 2.

## 4. Aptamers Used in Breast Cancer Therapy

In breast cancer therapy, aptamers have established themselves as highly precise therapeutic tools owing to their ability to act as antagonists of target molecules or as vehicles for ligand-directed targeting, that is, the selective delivery of therapeutic agents to tumor cells. This principle is based on the affinity and specificity of aptamers for proteins or receptors overexpressed in malignant cells, guiding drugs, nucleic acids, or nano-cargos directly to the tumor site and reducing systemic exposure [13]. Through SELEX technologies, aptamers have been developed against relevant targets such as HER2, EpCAM, NCL, and receptors associated with tumor stem cells, allowing for the blockage of oncogenic signaling pathways or serving as delivery vectors for chemotherapeutics, interfering RNAs, nanoparticles, or photosensitizers. These strategies combine direct molecular inhibition with modulation of the tumor and immune microenvironment, advancing towards more precise, personalized therapies with lower toxicity.

### 4.1. Aptamers as Therapeutic Agents and Specific Target Modulators

Aptamers can act as direct antagonists of proteins critical for tumor survival and progression. The AS1411 aptamer, directed against nucleolin (NCL), inhibits cell proliferation and enhances radiosensitivity when combined with gold nanoparticles, being one of the first aptamers evaluated clinically [55,56]. Likewise, Axl-148b blocks tumor progression in breast cancer and melanoma [45], while an RNA aptamer against osteopontin (OPN) suppresses growth and metastasis in MDA-MB-231 cells [44]. At the cutting edge, Aptamer–PROTAC conjugates facilitate the selective degradation of oncogenic proteins via targeted ubiquitination [34], and the Q10 aptamer, obtained by Exo-SELEX from metastatic exosomes, reduces angiogenesis and pulmonary metastasis without systemic toxicity [12]. Aptamers targeting VRK1 and PAI-1 inhibit proliferation and migration [44,48], and apMNKQ2 against kinase MNK1b blocks protein translation linked to invasive phenotypes [34]. Blocking NCL also modulates oncogenic microRNAs, reducing tumor aggressiveness [53]. These results, although mostly preclinical, consolidate the value of aptamers as direct agents with high specificity, low immunogenicity, and excellent chemical reproducibility. However, their in vivo half-life is usually short (minutes to a few hours), and rapid renal clearance (<30 kDa) limits their therapeutic efficacy, necessitating PEGylation or nanoparticle formulations to prolong circulation and biodistribution.

### 4.2. Aptamers in Chemotherapeutic Drug Delivery Systems

Aptamers are widely used in targeted delivery systems for chemotherapeutic agents, increasing efficacy and reducing systemic toxicity. They have been coupled to liposomes, polymeric nanoparticles, micelles, and functionalized mesoporous silica structures targeting MUC1, HER2, EGFR, or NCL to transport drugs such as doxorubicin, docetaxel, paclitaxel, or cisplatin [32,65,66]. Notable examples include dual polymeric micelles (SRL2–TA1) with docetaxel, which induce apoptosis and an antimetastatic effect [66], and anti-MUC1–DOX mesoporous nanoparticles with controlled release in MCF7 cells [57,58]. Combinations with paclitaxel, epirubicin, gemcitabine, or triptolide have produced multifunctional systems that overcome drug resistance, such as magnetic nanoparticles loaded with paclitaxel or selenium with epirubicin and the NAS-24 aptamer [11,47]. Anti-EGFR polymeric nanocarriers enhance tumor accumulation of cisplatin [65]. Finally, self-assembled DNA structures like nanotrenes and nanobarrels conjugated with AS1411 enable multiple payloads and synergistic intracellular release [36]. Collectively, these systems exhibit a clear relationship between molecular design and therapeutic benefit, though clinical parameters such as biodistribution, maximum tolerated dose, and tumor retention still need to be evaluated (TRL 4–5).

### 4.3. Combined Therapies with Chemotherapy, Radiotherapy, and Immunotherapy

Combined therapies integrating aptamers with traditional modalities seek therapeutic synergies and toxicity reduction. In chemotherapy, aptamer–siRNA conjugates allow simultaneous co-delivery of agents like doxorubicin, paclitaxel [67], and cisplatin, highlighting chimeras targeting EGFR/HER2/HER3 or EpCAM–siRNA, which reduce survivin expression and tumor stem cells [59]. Cationic liposomes functionalized with aptamers that co-deliver paclitaxel and anti-PLK1 siRNA synergistically inhibit tumor growth in vivo [54]. In radiotherapy, the conjugation of AS1411 with gold nanoparticles increases the nuclear deposition of radiation, intensifying tumor damage [56], while anti-PD-L1 hafnium oxide nanoparticles combine radiosensitization and near-infrared imaging [9]. In immunotherapy, anti-PD-L1 aptamers produce dual effects of immunosuppressive blockade and cytotoxicity when combined with chemotherapy; others, coupled to NK cells, increase selective cytotoxicity in triple-negative breast cancer [8,11]. Aptamer–PROTAC conjugates expand the selective degradation of immunosuppressive proteins [34]. Clinically, only a few aptamers (pegaptanib, NOX-A12) have reached early clinical stages in other tumors, highlighting the need for comparative trials to validate efficacy and safety in breast cancer (TRL 5–6).

### 4.4. Other Therapeutic Modalities

Aptamers are also applied in physical therapies such as photothermal and photodynamic therapy, directing photoactive agents with high specificity and minimal toxicity [60,61]. An overview of representative aptamer-guided photo-therapeutic strategies is provided in Table 3.

Biomimetic platforms based on DNA and metal–organic frameworks (MOFs) conjugated with aptamers respond to physiological stimuli such as pH, ATP, or microRNAs, releasing their cargo in the tumor microenvironment [12,40,66]. These include pH- and miRNA-sensitive DNA-MOF systems, dual ATP/pH nanoparticles, and targeted micelles that release doxorubicin at metastatic sites. Moreover, self-assembled structures like aptamer–DOX nanotrenes achieve efficient and selective drug delivery in tumor stem cells [36]. These platforms attain high levels of spatial and temporal control (TRL 4–5), though challenges in tissue penetration and thermal dissipation remain.

**Figure 4:**
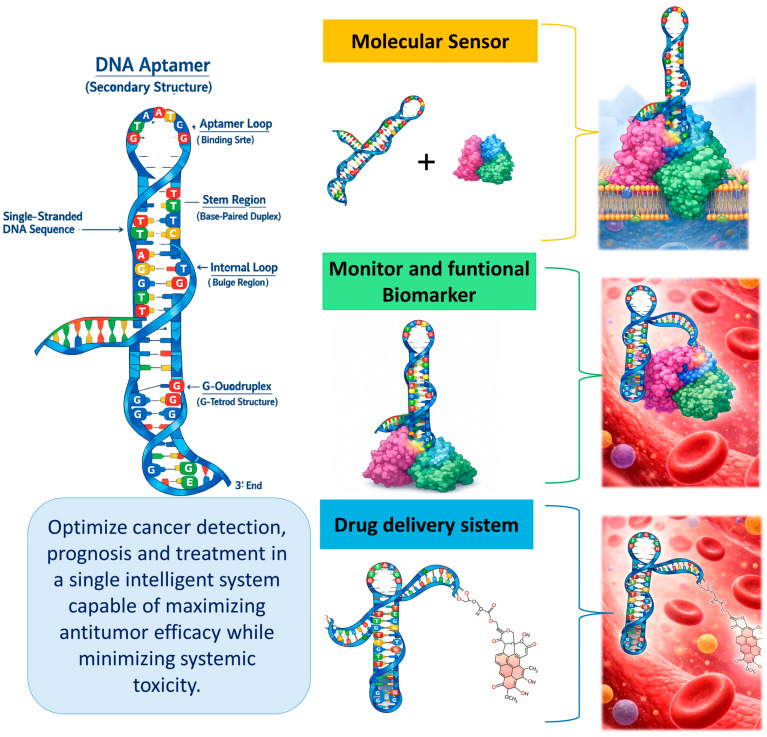
Multifunctional role of aptamers in effective theragnostic. Schematic representation of aptamers as central components of intelligent theragnostic systems. The second structure in 2D of the representative aptamers identifies the stem region, internal loop and single-stranded regions. Acting simultaneously as molecular sensors, monitoring biomarkers, and active targeting agents, aptamers bridge diagnostic and therapeutic functions within a single construct. This multifunctionality enables real-time monitoring, precise tumor localization, and controlled therapeutic delivery, optimizing cancer detection, prognosis, and treatment while minimizing systemic toxicity and off-target effects [37,39,43,52,55,63].

Overall, aptamer-based therapies integrate molecular specificity, chemical modularity, and compatibility with nanotechnology, positioning them as cornerstones of precision medicine. Consistent with this concept, Figure 4 summarizes the multifunctional contribution of aptamers to effective theranostics, depicting their role as central building blocks within intelligent diagnostic–therapeutic platforms. However, the lack of clinical studies, pharmacokinetic limitations, and variability of in vivo response mean caution is required before their widespread therapeutic adoption. Their future will depend on optimizing stability, biodistribution, and comparative validation against antibodies and existing biological platforms.

## 5. Perspectives and Challenges in Latin America

**Figure 5:**
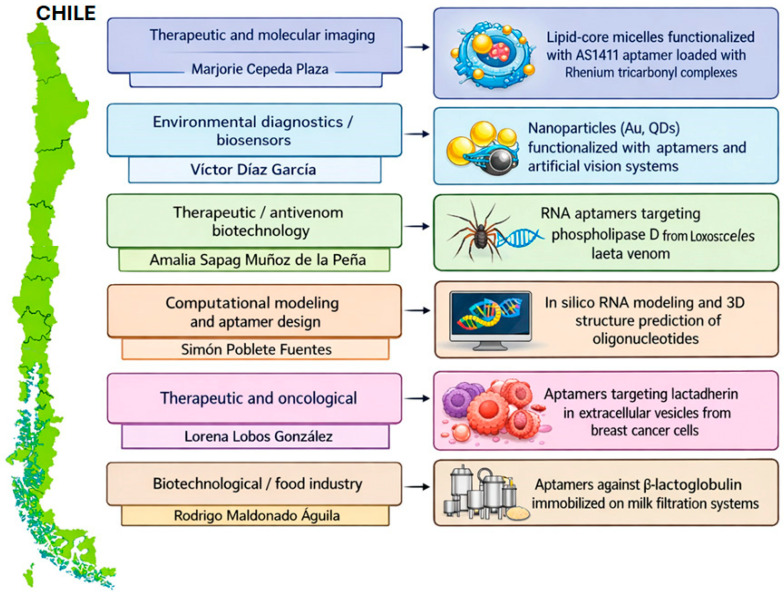
Overview of Chilean scientific contributions to aptamer-based research. Representation of leading research lines in Chile focused on aptamer technology and its multidisciplinary applications. The figure highlights key investigators and thematic areas that exemplify the regional development of aptamer science. Marjorie Cepeda Plaza explores therapeutic micellar systems functionalized with the AS1411 aptamer for molecular imaging and targeted delivery. Víctor Díaz García advances environmental diagnostics through nanoparticle-based biosensors. Amalia Sapag Muñoz de la Peña pioneers RNA aptamers with antivenom and biomedical potential, while Simón Poblete Fuentes applies computational modeling and in silico structure prediction to rational aptamer design. Lorena Lobos González leads oncological research targeting lactadherin in extracellular vesicles from breast cancer cells, and Rodrigo Maldonado Águila develops aptamer-functionalized systems for biotechnological and food industry applications. Together, these initiatives demonstrate Chile’s growing leadership in translational biotechnology, bridging molecular design, nanotechnology, and clinical innovation to position Latin America as a relevant contributor to the global aptamer field [67].

Research on aptamers has grown steadily in Latin America, with Chile, Argentina, and Mexico serving as key centers for the development of the diagnosis, prognosis, and therapy of breast cancer and other diseases. In Chile, the first “Aptamers in Chile 2024” meeting (organized by Dr. Amalia Sapag, University of Chile) showcased a well-established scientific community. Notable contributions include the work of Dr. Marjorie Cepeda (UNAB/U. de Chile) on lipid micelles with the AS1411 aptamer for imaging and targeted therapy; Dr. Víctor Díaz (USS) on biosensors with nanoparticles; Dr. Simón Poblete (Ciencia & Vida Foundation) on RNA structural modeling; and the “Apta-TumorStop” project (Dr. Lorena Lobos-González, U. de Chile/ACCDiS), which managed to reduce tumorigenesis by blocking lactadherin in extracellular vesicles. Additionally, Dr. Rodrigo Maldonado (USS/CECs) demonstrated food applications through aptamers designed to eliminate β-lactoglobulin, reflecting the technological versatility of the Chilean ecosystem. An overview of the Chilean aptamer landscape and representative initiatives is presented in Figure 5.

In Argentina, collaboration between CONICET and national universities has spurred the development of aptasensors and nano-aptamer platforms. Dr. Laura Raiger’s group (CONICET-UBA) has designed electrochemical and optical biosensors for contaminants and clinical biomarkers, while the NanoBioSensors-INQUIMAE team led by Dr. Michael López works on the conjugation of aptamers with nanomaterials such as graphene, gold, and zinc oxide, applicable to molecular diagnosis and personalized medicine.

Mexico has strengthened its work on structural design and nanobiotechnology applied to aptamers. CICESE has modeled the interaction of the anti-MUC1 aptamer with its epitope; CINVESTAV leads SELEX selection programs; UNAM develops G-quadruplex aptamers with anti-angiogenic potential and metal conjugates for molecular detection; and UANL works on nanosystems targeting HER2, aimed at precision medicine. Despite these advances, the region faces translational gaps: intermittent funding, uneven infrastructure, lack of clinical trials, and regulatory frameworks adapted to oligonucleotide-based therapies. There is also an ongoing need to standardize analytical parameters—sensitivity, specificity, and reproducibility—to compare prototypes with international platforms.

Taken together, the progress in Chile, Argentina, and Mexico demonstrates that the region has the scientific capacity to join precision medicine based on aptamers. The immediate challenge is to transform experimental advances into robust clinical evidence through multicenter consortia, shared biobanks, and joint validation and regulatory strategies, propelling Latin America toward an active role in translational biotechnology.

Despite the significant scientific progress achieved in Latin America, the clinical translation of aptamer-based technologies still faces important limitations. These include restricted in vivo stability of oligonucleotides, potential immunogenicity associated with chemical modifications or nanocarriers, and challenges in scaling up SELEX protocols under standardized and regulatory-compliant conditions. In addition, fragmented funding schemes, limited access to GMP-grade manufacturing, and the scarcity of multicenter preclinical and clinical studies further delay clinical validation. In this context, regional collaborative networks represent a critical opportunity to overcome these translational barriers. The establishment of shared SELEX platforms, centralized biobanks, harmonized preclinical models, and joint regulatory strategies could significantly accelerate validation and scalability. Moreover, interdisciplinary consortia integrating basic scientists, clinicians, engineers, and regulatory experts would enable the transition from proof-of-concept studies to clinically relevant applications, positioning Latin America not only as a generator of innovative aptamer technologies but also as an active contributor to their translational implementation.

## 6. Conclusions

Aptamers have emerged as key tools in contemporary biomedicine, especially in the diagnosis and treatment of breast cancer. Their nature as DNA or RNA oligonucleotides gives them unique advantages over monoclonal antibodies, including low synthesis cost, high specificity, chemical reproducibility, and ease of structural modification. These properties, along with advancements in SELEX technologies and the ability to incorporate stabilizing chemical modifications, have propelled their development as versatile molecular platforms capable of integration into biosensors, imaging systems, targeted therapies, and theranostic nanodevices.

In diagnosis and prognosis, aptamers have enabled the detection of relevant biomarkers such as HER2, MUC1, EpCAM, PD-L1, and nucleolin through biosensors, liquid biopsies, and high-resolution optical technologies. These strategies make early disease identification and molecular stratification of patients possible, improving clinical precision and monitoring therapeutic response. In therapy, aptamers act both as direct inhibitors of oncogenic pathways and as components in controlled delivery systems for drugs and nucleic acids. Conjugates such as aptamer–drug, aptamer–siRNA, and nano-aptamer structures have demonstrated preclinical efficacy in aggressive subtypes like triple-negative breast cancer, validating their potential in personalized medicine.

Despite these advances, challenges remain that limit their clinical translation: short half-life, rapid renal clearance, potential immunogenicity, and lack of multicenter validation. In this context, Latin America—with Chile, Argentina, and Mexico as leaders—has driven significant growth in translational research, biosensors, and structural design of aptamers, although regulatory and infrastructure gaps persist. The future of the field will depend on integrating artificial intelligence, nanotechnology, and systems pharmacology to optimize in vivo stability and efficacy. Overcoming these challenges will enable aptamers to be established as clinically approved agents and leading figures in 21st-century precision medicine.

## Figures and Tables

**Table 1:** General comparison between aptamers and therapeutic platforms against breast cancer.

	Aptamers (Gene Therapy) [18,19]	Antibodies (Immunotherapy) [20,21]	Oligonucleotides (Gene Therapy) [22,23]	Peptides (Proteomics) [24,25]
Immunogenicity	Low immunogenicity: suitable for repeated dosing.	May be immunogenic.	Generally low immunogenicity; design-dependent.	Moderate immunogenicity.
Cost	Low cost: scalable chemical synthesis.	High cost: requires cell culture and complex purification.	Medium cost: cheaper than proteins but typically more expensive than aptamers.	Medium cost: cheaper than proteins but less stable.
Specificity	High specificity: nanomolar–picomolar affinity.	Very high specificity: foundation of targeted therapies (e.g., trastuzumab).	High specificity: sequence-defined targeting.	High specificity but rapid degradation.
Half-life	Short to medium half-life: can be improved via chemical modifications (e.g., PEGylation).	Long half-life, especially with IgG.	Short to medium half-life: formulation-dependent.	Short half-life: rapidly degraded in blood.

**Table 2:** Classification of aptamer systems used in therapeutics.

Aptamer System Structure	Target	Mechanism of Uptake	Vehicle	Therapy	Reference
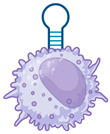	Unidentified surface target(s) on poorly differentiated cancer cells	Extracellular interaction	NK-92	Cell therapy	[8]
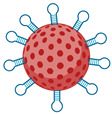	PD-L1	Receptor-mediated endocytosis	Mesoporous Hafnium Oxide Nanoparticles, MHNs	Rx: HfO2	[9]
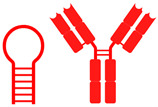	PDGFRβ	Extracellular interaction	Conjunted administation	anti-PDL1	[11]
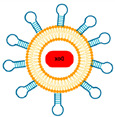	Surface proteins of the 4T1 cell line	Macropinocytosis/Receptor-mediated endocytosis	DOTAP + DOPE	Qx: Doxorubicin	[32]
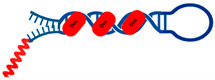	CD44	Receptor-mediated endocytosis	N/A	Rx: Doxorubicin Inhibited AKT peptide	[36]
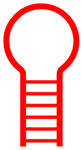	NCL	Macropinocytosis/Receptor-mediated endocytosis	Stand Alone	Stand Alone	[51]
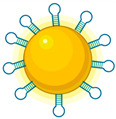	Nucleolin	Receptor-mediated endocytosis	Gold Nano Particles (GNPs)	Rx: Au	[52]
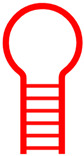	Osteopontin	Macropinocytosis/Receptor-mediated endocytosis	Stand Alone	Stand Alone	[54]
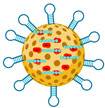	MUC1	Receptor-mediated endocytosis	Mesoporous silica MCM-41	Qx: Doxorubicin	[55]
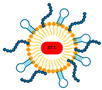	CD44	Macropinocytosis/Receptor-mediated endocytosis	Poly β-amino ester (PAE)	Qx: Docetaxel	[56]
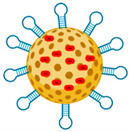	HER2	Diffusion/Receptor-mediated endocytosis	Mesoporous silica nanoparticles (MSNPs)	Qx: Doxorubicin	[57]
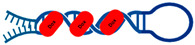	ABCG2	Diffusion/Receptor-mediated endocytosis	N/A	Qx: Doxorubicin	[58]
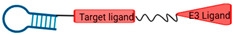	Nucleolin	Receptor-mediated endocytosis	N/A	PROTAC to BET proteins	[59]
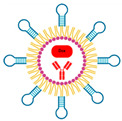	Nucleolin	Receptor-mediated endocytosis	TiO_2_ + Polidopamine (PDA)	Qx: Doxorubicin/anti-FOXM1	[60]
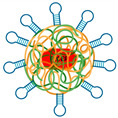	EGFR	Receptor-mediated endocytosis	F8BT Copolymer	Rx: PtOEP	[61]
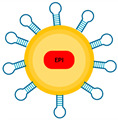	MUC1	Receptor-mediated endocytosis	Selenium nanoparticles (SeNPs)	Rx: Epirubicin/Anti-vimentin aptamer	[62]
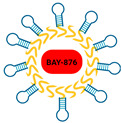	CTLA-4 and PDL1	Receptor-mediated endocytosis	Poly β-amino ester (PAE)	BAY-876 inh glut1	[63]
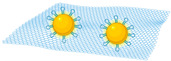	MUC1	Receptor-mediated endocytosis	Graphene Oxide	Rx: Au	[64]

**Table 3:** Chimerical aptamer systems for target–ligand gene therapy.

Aptamer System Structure	Target	Mechanism of Uptake	Gene Therapy Type	Reference
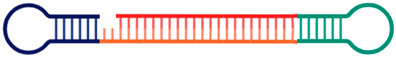	HER2 HER3	Receptor-mediated endocytosis	(siRNA) *EGFR*	[59]
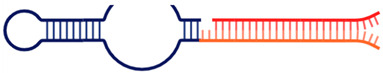	EpCAM	Receptor-mediated endocytosis	(siRNA) *Plk1*	[43]
	AXL	Receptor-mediated endocytosis	(miRNA) *ITGA5A*, *LCAM*	[45]
	EGFR	Receptor-mediated endocytosis	(Anti-miRNA) miR-21 (PTEN suppressor)	[62]
	HER2	Receptor-mediated endocytosis	(siRNA) *XBP1*	[63]
	EpCAM	Receptor-mediated endocytosis	(siRNA) *Upf2*, *Parp1*, *Cd47* and *Mcl1*	[64]

## Data Availability

The original contributions presented in this study are included in the article. Further inquiries can be directed to the corresponding author.

## Abbreviations

**Breast cancer subtypes and clinical markers**	
TNBC	Triple-Negative Breast Cancer
HER2	Human Epidermal Growth Factor Receptor 2
ER	Estrogen Receptor
PR	Progesterone Receptor
**Liquid biopsy, vesicles and imaging**	
CTC(s)	Circulating Tumor Cell(s)
EV(s)	Extracellular Vesicle(s)
SERS	Surface-Enhanced Raman Spectroscopy
SECT	Sequential Emission Computed Tomography
SPECT	Single-Photon Emission Computed Tomography
MRI	Magnetic Resonance Imaging
**Tumor biomarkers and receptors**	
MUC1	Mucin 1
EpCAM	Epithelial Cell Adhesion Molecule
PD-L1	Programmed Death-Ligand 1
PDGFRβ	Platelet-Derived Growth Factor Receptor Beta
AIB1	Amplified in Breast Cancer 1
NCL	Nucleolin
OPN	Osteopontin
VRK1	Vaccinia-Related Kinase 1
MNK1b	MAP Kinase-Interacting Kinase 1b
EGFR	Epidermal Growth Factor Receptor
AXL	AXL Receptor Tyrosine Kinase
HER3	Human Epidermal Growth Factor Receptor 3 (ERBB3)
**Aptamer platforms and molecular tools**	
SELEX	Systematic Evolution of Ligands by Exponential Enrichment
Cell-SELEX	Cell-based SELEX
Exo-SELEX	Exosome-guided SELEX
AS1411	Nucleolin-Binding Aptamer
Q10	Anti-metastatic Exo-SELEX Aptamer
Axl-148b	Anti-Axl Aptamer
**Therapeutic systems and nanotechnology**	
siRNA	Small Interfering RNA
PROTAC	Proteolysis-Targeting Chimera
APC	Aptamer–PROTAC Conjugate
DOX	Doxorubicin
PTX	Paclitaxel
DOC	Docetaxel
PEG	Polyethylene Glycol
MOF	Metal–Organic Framework
ATP	Adenosine Triphosphate
NIR	Near-Infrared
DOTAP	1,2-dioleoyl-3-(trimethylammonium)propane
DOPE	1,2-dioleoyl-sn-glycero-3-phosphoethanolamine
**Therapies**	
Rx	Radiotherapy
Qx	Chemotherapy
**Cell lines**	
MCF-7	Human Breast Adenocarcinoma Cells
SK-BR-3	HER2-Positive Breast Cancer Cells
MDA-MB-231	Triple-Negative Breast Cancer Cells
**Nanomaterials**	
Fe_3_O_4_	Magnetite Nanoparticles
AuNPs	Gold Nanoparticles
GNPs	Gold Nanoparticles
Ag–Au	Silver–Gold Nanostructures
QDs	Quantum Dots
DNA-Au	DNA–Gold Nanomachine
MSNPs	Mesoporous Silica Nanoparticles
MCM-41 (*o MCM41*)	Mobil Composition of Matter No. 41 (mesoporous silica)
SeNPs	Selenium Nanoparticles
PDA	Polydopamine
PAE	Poly(β-amino ester)
BET	Brunauer–Emmett–Teller (BET) theory/surface area analysis
PtOEP	Platinum(II) Octaethylporphyrin
PdOEP	Palladium(II) Octaethylporphyrin
FBBT	poly(9,9-dioctylfluorene-alt-benzothiadiazole)
**Signaling, resistance and survival markers (genes/proteins)**	
FOXM1	Forkhead Box M1
AKT	Protein Kinase B (AKT)
ABCG2	ATP-Binding Cassette Subfamily G Member 2
CD44	Cluster of Differentiation 44
ITGA5 (*en tu lista: ITGA5A*)	Integrin Subunit Alpha 5
LCAM (L-CAM)	Liver Cell Adhesion Molecule (E-cadherin/CDH1)
UPF2	UPF2 Regulator of Nonsense-Mediated mRNA Decay
PARP1	Poly(ADP-ribose) Polymerase 1
CD47	Cluster of Differentiation 47
MCL1	Myeloid Cell Leukemia 1
PTEN	Phosphatase and Tensin Homolog
PLK1	Polo-Like Kinase 1
**Others**	
miRNA	MicroRNA
TME	Tumor Microenvironment
EPR	Enhanced Permeability and Retention Effect

## References

[1] World Health Organization (2024). Breast Cancer.

[2] Sung H., Ferlay J., Siegel R.L., Laversanne M., Soerjomataram I., Jemal A., Bray F. (2021). Global Cancer Statistics 2020: GLOBOCAN Estimates of Incidence and Mortality Worldwide for 36 Cancers in 185 Countries. CA Cancer J. Clin..

[3] Bianchini G., Balko J.M., Mayer I.A., Sanders M.E., Gianni L. (2016). Triple-Negative Breast Cancer: Challenges and Opportunities of a Heterogeneous Disease. Nat. Rev. Clin. Oncol..

[4] Tuerk C., Gold L. (1990). Systematic Evolution of Ligands by Exponential Enrichment: RNA Ligands to Bacteriophage T4 DNA Polymerase. Science.

[5] Ellington A.D., Szostak J.W. (1990). In Vitro Selection of RNA Molecules That Bind Specific Ligands. Nature.

[6] Sefah K., Shangguan D., Xiong X., O’Donoghue M.B., Tan W. (2010). Development of DNA Aptamers Using Cell-SELEX. Nat. Protoc..

[7] Amero P., Lokesh G.L.R., Chaudhari R.R., Cardenas-Zuniga R., Schubert T., Attia Y.M., Montalvo-Gonzalez E., Elsayed A.M., Ivan C., Wang Z. (2021). Conversion of RNA Aptamer into Modified DNA Aptamers Provides for Prolonged Stability and Enhanced Antitumor Activity. J. Am. Chem. Soc..

[8] Chen Z., Zeng Z., Wan Q., Liu X., Qi J., Zu Y. (2022). Targeted Immunotherapy of Triple-Negative Breast Cancer by Aptamer-Engineered NK Cells. Biomaterials.

[9] Wei M., Shen X., Fan X., Li J., Bai J. (2023). PD-L1 Aptamer-Functionalized Degradable Hafnium Oxide Nanoparticles for near Infrared-II Diagnostic Imaging and Radiosensitization. Front. Bioeng. Biotechnol..

[10] Maradani B.S., Parameswaran S., Subramanian K. (2022). Development and Characterization of DNA Aptamer against Retinoblastoma by Cell-SELEX. Sci. Rep..

[11] Camorani S., Passariello M., Agnello L., Esposito S., Collina F., Cantile M., Di Bonito M., Ulasov I.V., Fedele M., Zannetti A. (2020). Aptamer Targeted Therapy Potentiates Immune Checkpoint Blockade in Triple-Negative Breast Cancer. J. Exp. Clin. Cancer Res..

[12] Wang Q., Miao Z., Sun S., Ma Y., Chen Z., Li Y., Ba W., Liang M., Fang J., Li W. (2025). Discovery of a Novel DNA Aptamer for Impeding Tumor Metastasis by Blocking the Functional Activity of Target Protein on Exosome. Chem. Eng. J..

[13] Zhou J., Rossi J. (2017). Aptamers as Targeted Therapeutics: Current Potential and Challenges. Nat. Rev. Drug Discov..

[14] Ni X., Castanares M., Mukherjee A., Lupold S.E. (2011). Nucleic Acid Aptamers: Clinical Applications and Promising New Horizons. Curr. Med. Chem..

[15] Shraim A.S., Abdel Majeed B.A., Al-Binni M.A., Hunaiti A. (2022). Therapeutic Potential of Aptamer–Protein Interactions. ACS Pharmacol. Transl. Sci..

[16] Edwards A.N., Iannucci A.N., VanDenBerg J., Kesti A., Rice T., Sethi S., Dhakal S., Yangyuoru P.M. (2024). G-Quadruplex Structure in the ATP-Binding DNA Aptamer Strongly Modulates Ligand Binding Activity. ACS Omega.

[17] Gao F., Yin J., Chen Y., Guo C., Hu H., Su J. (2022). Recent Advances in Aptamer-Based Targeted Drug Delivery Systems for Cancer Therapy. Front. Bioeng. Biotechnol..

[18] Mahmoudian F., Ahmari A., Shabani S., Sadeghi B., Fahimirad S., Fattahi F. (2024). Aptamers as an Approach to Targeted Cancer Therapy. Cancer Cell Int..

[19] Sun H., Zhu X., Lu P.Y., Rosato R.R., Tan W., Zu Y. (2014). Oligonucleotide Aptamers: New Tools for Targeted Cancer Therapy. Mol. Ther.—Nucleic Acids.

[20] Zahavi D., Weiner L. (2020). Monoclonal Antibodies in Cancer Therapy. Antibodies.

[21] Nelson A.L., Dhimolea E., Reichert J.M. (2010). Development Trends for Human Monoclonal Antibody Therapeutics. Nat. Rev. Drug Discov..

[22] Zhu Y., Zhu L., Wang X., Jin H. (2022). RNA-Based Therapeutics: An Overview and Prospectus. Cell Death Dis..

[23] Çakan E., Lara O.D., Szymanowska A., Bayraktar E., Chavez-Reyes A., Lopez-Berestein G., Amero P., Rodriguez-Aguayo C. (2024). Therapeutic Antisense Oligonucleotides in Oncology: From Bench to Bedside. Cancers.

[24] Fosgerau K., Hoffmann T. (2015). Peptide Therapeutics: Current Status and Future Directions. Drug Discov. Today.

[25] Vlieghe P., Lisowski V., Martinez J., Khrestchatisky M. (2010). Synthetic Therapeutic Peptides: Science and Market. Drug Discov. Today.

[26] Domsicova M., Korcekova J., Poturnayova A., Breier A. (2024). New Insights into Aptamers: An Alternative to Antibodies in the Detection of Molecular Biomarkers. Int. J. Mol. Sci..

[27] Agnello L., Camorani S., Fedele M., Cerchia L. (2021). Aptamers and Antibodies: Rivals or Allies in Cancer Targeted Therapy?. Explor. Target. Anti-Tumor Ther..

[28] Wu P., Gao Y., Zhang H., Cai C. (2012). Aptamer-Guided Silver–Gold Bimetallic Nanostructures with Highly Active Surface-Enhanced Raman Scattering for Specific Detection and Near-Infrared Photothermal Therapy of Human Breast Cancer Cells. Anal. Chem..

[29] Chinnappan R., Al Faraj A., Abdel Rahman A.M., Abu-Salah K.M., Mouffouk F., Zourob M. (2020). Anti-VCAM-1 and Anti-IL4Rα Aptamer-Conjugated Super Paramagnetic Iron Oxide Nanoparticles for Enhanced Breast Cancer Diagnosis and Therapy. Molecules.

[30] Hosseine M., Naghib S.M., Khodadadi A. (2024). Label-Free Electrochemical Biosensor Based on Green-Synthesized Reduced Graphene Oxide/Fe_3_O_4_/Nafion/Polyaniline for Ultrasensitive Detection of SKBR3 Cell Line of HER2 Breast Cancer Biomarker. Sci. Rep..

[31] An Y., Wu J., Yang B., Zhu Z., Gao M., Yu C., Yang C.J. (2015). Selection and Application of DNA Aptamer Against Oncogene Amplified in Breast Cancer 1. J. Mol. Evol..

[32] Song Y., Zhu Z., An Y., Zhang W., Zhang H., Liu D., Yu C., Duan W., Yang C.J. (2013). Selection of DNA Aptamers against Epithelial Cell Adhesion Molecule for Cancer Cell Imaging and Circulating Tumor Cell Capture. Anal. Chem..

[33] Albanese C.M., Suttapitugsakul S., Perati S., McGown L.B. (2018). A Genome-Inspired, Reverse Selection Approach to Aptamer Discovery. Talanta.

[34] Santos Do Carmo F., Ricci-Junior E., Cerqueira-Coutinho C., Albernaz M.D.S., Bernardes E.S., Missailidis S., Santos-Oliveira R. (2016). Anti-MUC1 Nano-Aptamers for Triple-Negative Breast Cancer Imaging by Single-Photon Emission Computed Tomography in Inducted Animals: Initial Considerations. Int. J. Nanomed..

[35] He Y., Wang M., Fu M., Yuan X., Luo Y., Qiao B., Cao J., Wang Z., Hao L., Yuan G. (2020). Iron(II) Phthalocyanine Loaded and AS1411 Aptamer Targeting Nanoparticles: A Nanocomplex for Dual Modal Imaging and Photothermal Therapy of Breast Cancer. Int. J. Nanomed..

[36] Bruno J.G. (2010). Aptamer–Biotin–Streptavidin–C1q Complexes Can Trigger the Classical Complement Pathway to Kill Cancer Cells. Vitro Cell. Dev. Biol.—Anim..

[37] Hua X., Zhou Z., Yuan L., Liu S. (2013). Selective Collection and Detection of MCF-7 Breast Cancer Cells Using Aptamer-Functionalized Magnetic Beads and Quantum Dots Based Nano-Bio-Probes. Anal. Chim. Acta.

[38] Shen C., Liu S., Li X., Yang M. (2019). Electrochemical Detection of Circulating Tumor Cells Based on DNA Generated Electrochemical Current and Rolling Circle Amplification. Anal. Chem..

[39] Mohammadi S., Salimi A., Hamd-Ghadareh S., Fathi F., Soleimani F. (2018). A FRET Immunosensor for Sensitive Detection of CA 15-3 Tumor Marker in Human Serum Sample and Breast Cancer Cells Using Antibody Functionalized Luminescent Carbon-Dots and AuNPs-Dendrimer Aptamer as Donor-Acceptor Pair. Anal. Biochem..

[40] Zhang Y., Lu Y., Wang F., An S., Zhang Y., Sun T., Zhu J., Jiang C. (2017). ATP/pH Dual Responsive Nanoparticle with _D_-[des-Arg^10^]Kallidin Mediated Efficient In Vivo Targeting Drug Delivery. Small.

[41] Luo J., Liang D., Li X., Deng L., Wang Z., Yang M. (2020). Aptamer-Based Photoelectrochemical Assay for the Determination of MCF-7. Microchim. Acta.

[42] Esposito C.L., Quintavalle C., Ingenito F., Rotoli D., Roscigno G., Nuzzo S., Thomas R., Catuogno S., De Franciscis V., Condorelli G. (2021). Identification of a Novel RNA Aptamer That Selectively Targets Breast Cancer Exosomes. Mol. Ther. Nucleic Acids.

[43] Gilboa-Geffen A., Hamar P., Le M.T.N., Wheeler L.A., Trifonova R., Petrocca F., Wittrup A., Lieberman J. (2015). Gene Knockdown by EpCAM Aptamer–siRNA Chimeras Suppresses Epithelial Breast Cancers and Their Tumor-Initiating Cells. Mol. Cancer Ther..

[44] Mi Z., Guo H., Russell M.B., Liu Y., Sullenger B.A., Kuo P.C. (2009). RNA Aptamer Blockade of Osteopontin Inhibits Growth and Metastasis of MDA-MB231 Breast Cancer Cells. Mol. Ther..

[45] Quirico L., Orso F., Esposito C.L., Bertone S., Coppo R., Conti L., Catuogno S., Cavallo F., De Franciscis V., Taverna D. (2020). Axl-148b Chimeric Aptamers Inhibit Breast Cancer and Melanoma Progression. Int. J. Biol. Sci..

[46] Masoudi M., Taghdisi S.M., Hashemitabar G., Abnous K. (2024). Targeted Co-Delivery of FOXM1 Aptamer and DOX by Nucleolin Aptamer-Functionalized pH-Responsive Biocompatible Nanodelivery System to Enhance Therapeutic Efficacy against Breast Cancer: In Vitro and in Vivo. Drug Deliv. Transl. Res..

[47] Yu S., Zhou Y., Sun Y., Wu S., Xu T., Chang Y., Bi S., Jiang L., Zhu J. (2021). Endogenous mRNA Triggered DNA-Au Nanomachine for In Situ Imaging and Targeted Multimodal Synergistic Cancer Therapy. Angew. Chem. Int. Ed..

[48] Carrión-Marchante R., Frezza V., Salgado-Figueroa A., Pérez-Morgado M.I., Martín M.E., González V.M. (2021). DNA Aptamers against Vaccinia-Related Kinase (VRK) 1 Block Proliferation in MCF7 Breast Cancer Cells. Pharmaceuticals.

[49] Yin H., Xiong G., Guo S., Xu C., Xu R., Guo P., Shu D. (2019). Delivery of Anti-miRNA for Triple-Negative Breast Cancer Therapy Using RNA Nanoparticles Targeting Stem Cell Marker CD133. Mol. Ther..

[50] Lu M., Zhou L., Zheng X., Quan Y., Wang X., Zhou X., Ren J. (2015). A Novel Molecular Marker of Breast Cancer Stem Cells Identified by Cell-SELEX Method. Cancer Biomark..

[51] Lv J., Li S., Zhen X., Li D., Zhang N., Liu X., Han J., Bing T., Shangguan D. (2022). Characterization and Identification of Aptamers against CD49c for the Detection, Capture, and Release of Cancer Cells. ACS Appl. Bio Mater..

[52] Domenyuk V., Gatalica Z., Santhanam R., Wei X., Stark A., Kennedy P., Toussaint B., Levenberg S., Wang J., Xiao N. (2018). Poly-Ligand Profiling Differentiates Trastuzumab-Treated Breast Cancer Patients According to Their Outcomes. Nat. Commun..

[53] Pichiorri F., Palmieri D., De Luca L., Consiglio J., You J., Rocci A., Talabere T., Piovan C., Lagana A., Cascione L. (2013). In Vivo NCL Targeting Affects Breast Cancer Aggressiveness through miRNA Regulation. J. Exp. Med..

[54] Li M., Li S., Li Y., Li X., Yang G., Li M., Xie Y., Su W., Wu J., Jia L. (2022). Cationic Liposomes Co-Deliver Chemotherapeutics and siRNA for the Treatment of Breast Cancer. Eur. J. Med. Chem..

[55] Reyes-Reyes E.M., Teng Y., Bates P.J. (2010). A New Paradigm for Aptamer Therapeutic AS1411 Action: Uptake by Macropinocytosis and Its Stimulation by a Nucleolin-Dependent Mechanism. Cancer Res..

[56] Ghahremani F., Kefayat A., Shahbazi-Gahrouei D., Motaghi H., Mehrgardi M.A., Haghjooy-Javanmard S. (2018). AS1411 Aptamer-Targeted Gold Nanoclusters Effect on the Enhancement of Radiation Therapy Efficacy in Breast Tumor-Bearing Mice. Nanomedicine.

[57] Liu Z., Duan J.-H., Song Y.-M., Ma J., Wang F.-D., Lu X., Yang X.-D. (2012). Novel HER2 Aptamer Selectively Delivers Cytotoxic Drug to HER2-Positive Breast Cancer Cells in Vitro. J. Transl. Med..

[58] Charbgoo F., Alibolandi M., Taghdisi S.M., Abnous K., Soltani F., Ramezani M. (2018). MUC1 Aptamer-Targeted DNA Micelles for Dual Tumor Therapy Using Doxorubicin and KLA Peptide. Nanomed. Nanotechnol. Biol. Med..

[59] Yu X., Ghamande S., Liu H., Xue L., Zhao S., Tan W., Zhao L., Tang S.-C., Wu D., Korkaya H. (2018). Targeting EGFR/HER2/HER3 with a Three-in-One Aptamer-siRNA Chimera Confers Superior Activity against HER2+ Breast Cancer. Mol. Ther.—Nucleic Acids.

[60] Yang L., Tseng Y.-T., Suo G., Chen L., Yu J., Chiu W.-J., Huang C.-C., Lin C.-H. (2015). Photothermal Therapeutic Response of Cancer Cells to Aptamer–Gold Nanoparticle-Hybridized Graphene Oxide under NIR Illumination. ACS Appl. Mater. Interfaces.

[61] Ibarra L.E., Camorani S., Agnello L., Pedone E., Pirone L., Chesta C.A., Palacios R.E., Fedele M., Cerchia L. (2022). Selective Photo-Assisted Eradication of Triple-Negative Breast Cancer Cells through Aptamer Decoration of Doped Conjugated Polymer Nanoparticles. Pharmaceutics.

[62] Zhang L., Mu C., Zhang T., Wang Y., Wang Y., Fan L., Liu C., Chen H., Shen J., Wei K. (2020). Systemic Delivery of Aptamer-Conjugated XBP1 siRNA Nanoparticles for Efficient Suppression of HER2+ Breast Cancer. ACS Appl. Mater. Interfaces.

[63] Sociedad de Biología de Chile Aptámeros En Chile 2024. Scientific Event Held on 26 November 2024, Santiago, Chile. https://www.biologiachile.cl/2024/10/14/aptameros-en-chile/.

[64] Zhang Q., Ma R., Zhang Y., Zhao J., Wang Y., Xu Z. (2023). Dual-aptamer-assisted ratiometric SERS biosensor for ultrasensitive and precise identification of breast cancer exosomes. ACS Sens..

[65] Esmaeili Y., Dabiri A., Mashayekhi F., Rahimmanesh I., Bidram E., Karbasi S., Rafienia M., Javanmard S.H., Ertas Y.N., Zarrabi A. (2024). Smart Co-Delivery of Plasmid DNA and Doxorubicin Using MCM-Chitosan-PEG Polymerization Functionalized with MUC-1 Aptamer against Breast Cancer. Biomed. Pharmacother..

[66] Taghipour Y.D., Zarebkohan A., Salehi R., Talebi M., Rahbarghazi R., Khordadmehr M., Khavandkari S., Badparvar F., Torchilin V.P. (2024). Enhanced Docetaxel Therapeutic Effect Using Dual Targeted SRL-2 and TA1 Aptamer Conjugated Micelles in Inhibition Balb/c Mice Breast Cancer Model. Sci. Rep..

[67] Kadkhoda J., Aghanejad A., Safari B., Barar J., Rasta S.H., Davaran S. (2022). Aptamer-conjugated gold nanoparticles for t argeted paclitaxel delivery and photothermal therapy in breast cancer. J. Drug Deliv. Sci. Technol..

